# Ongoing ecological and evolutionary consequences by the presence of transgenes in a wild cotton population

**DOI:** 10.1038/s41598-021-81567-z

**Published:** 2021-01-21

**Authors:** Valeria Vázquez-Barrios, Karina Boege, Tania Gabriela Sosa-Fuentes, Patricia Rojas, Ana Wegier

**Affiliations:** 1 Posgrado en Ciencias Biológicas, Instituto de Biología, Universidad Nacional Autónoma de México, Mexico City, Mexico; 2 Laboratorio de Genética de la Conservación, Jardín Botánico, Instituto de Biología, Universidad Nacional Autónoma de México, Mexico City, Mexico; 3 Departamento de Ecología Evolutiva, Instituto de Ecología, Universidad Nacional Autónoma de México, Mexico City, Mexico; 4 Red de Biodiversidad y Sistemática, Instituto de Ecología A.C., Xalapa, Veracruz Mexico

**Keywords:** Ecology, Evolution, Genetics, Plant sciences

## Abstract

After 25 years of genetically modified cotton cultivation in Mexico, gene flow between transgenic individuals and their wild relatives represents an opportunity for analysing the impacts of the presence of novel genes in ecological and evolutionary processes in natural conditions. We show comprehensive empirical evidence on the physiological, metabolic, and ecological effects of transgene introgression in wild cotton, *Gossypium hirsutum*. We report that the expression of both the *cry* and *cp4-epsps* genes in wild cotton under natural conditions altered extrafloral nectar inducibility and thus, its association with different ant species: the dominance of the defensive species *Camponotus planatus* in Bt plants, the presence of *cp4-epsps* without defence role of *Monomorium ebeninum* ants, and of the invasive species *Paratrechina longicornis* in wild plants without transgenes. Moreover, we found an increase in herbivore damage to *cp4-epsps* plants. Our results reveal the influence of transgene expression on native ecological interactions. These findings can be useful in the design of risk assessment methodologies for genetically modified organisms and the in situ conservation of *G. hirsutum* metapopulations.

## Introduction

The introgression of transgenes from economically important GM crops to their wild relatives^1^ (e.g. cotton^2,3^, sunflower^4^, rice^5^, and native papaya breeds^6^) has been frequently reported in the last decade within the natural distribution of the latter. However, the consequences of transgene expression in natural ecosystems remain unexplored, leading to uncertainty about its impact on local evolutionary and ecological processes. Research on this topic has been challenging largely because, while agro-biotechnological innovations move rapidly, the information required for adequate risk assessment is still limited^7^. However, knowledge on transgene introgression effects in natural conditions and in particularly in the species centre of origin can offer valuable information for in situ conservation of crop wild relatives (CWR).

The different disciplines interested in assessing the expected functions of genetically engineered genes have produced a fair amount of research in controlled environments, with the only purpose of applying the generated information to improving technological packages^8–10^. However, there is great uncertainty and limited evidence regarding transgene behaviour in wild genotypes that survive in natural conditions under a set of complex ecological challenges^7^. Consequently, studying transgenes in the wild represents an excellent opportunity to integrate a new stage of scientific knowledge, combining genetic, ecological, evolutionary, and environmental information.

Recently, several studies have looked for differences in genetic expression, fitness, and diversity in non-target organisms, between transgenic organisms and their isogenic lines, as well as between experimental parameters and settings (e.g. laboratory, crops fields, and greenhouse conditions)^11–14^. From these studies, we can infer that differences found in seed production and attraction of herbivores’ natural enemies are linked to the interaction of transgenes (i.e. *cry* and *cp4-epsps*). Such differences have been associated with defence mechanisms and/or main metabolic pathways involved in plant growth, development, and reproduction, such as the shikimate or the octadecanoid pathways^15,16^. As a consequence, the quantity and quality of metabolic products are modified, compromising not only plant performance but also their interactions with insects, bacteria, and soil fungi^17^.

To assess the consequences of transgenic expression in the absence of target pests and glyphosate as selection agents, we studied wild upland cotton (*Gossypium hirsutum* L.) and its associated ant communities in Mexico where the wild to domesticated complex exists. Upland cotton is a Mesoamerican native species in which introgression of transgenes conferring resistance to specific lepidopteran larvae (*Helicoverpa armigera, H. punctigera, Heliothis virescens, Pectinophora gossypiella*) (*Bt* or *cry* genes) and tolerance to glyphosate (*cp4-epsps*) have been detected, at its center of origin, in seven out of eight metapopulations (Baja California Sur, BCSM; North Pacific, NPM; Central Pacific, CPM; South Pacific, SPM; Gulf North, GNM; Gulf South, GSM; and Yucatán Peninsula, YPM)^2,3^. We examined the potential effects of introgressed transgenes on *G. hirsutum* production of extrafloral nectar, which constitutes a reward for defensive ants who patrol the plant to repel herbivores^18,19^. Assessing the ant-cotton interaction is relevant because ants are not-target insects of transgenic technology and their role is protecting cotton plants against herbivores^20^. Furthermore, in the coastal sand dunes where the studied populations are established, ants play important ecological functions acting as soil engineers and seed dispersers, influencing plant germination, reproductive success, and distribution patterns. To this end, we analyzed three wild cotton genotypes, two of which are introgressed plants with transgenes. All of them are growing in the species centre of origin: (1) wild genotypes without transgenes; (2) wild genotypes with *cry*; and (3) wild genotypes with *cp4-epsps* (hereafter W, W*cry*, and W*cp4-epsps*, respectively). We evaluated the inducibility of extrafloral nectar (EFN) after exogenous application of methyl jasmonate (MeJA), which has been widely used as a tool when studying plant reactions to stress and understanding the interactions between the elicitation of plant responses and their consequences for herbivores and beneficial insects^21^. We also measure the response of the ant community and herbivore damage associated with the three cotton genotypes.

## Material and methods

### Study site

This study was conducted in the costal dunes of the Ría Lagartos Biosphere Reserve (hereafter RL), Yucatan, Mexico. We located it in the stub zone, where wild cotton, *G. hirsutum,* is distributed^22^ (Table S1). We chose this site for the purpose of keeping track of the introgressive hybridisation between GM domesticated and wild plants^3^ within the RL, a place that allows long-term research without direct evidence of the anthropisation effect (i.e. land-use change).

### Experimental plants

To determine the presence of the herbicide-tolerant transgenes and those with insecticide effects within the studied population, in March 2018 we collected the foliar tissue of 61 cotton plants (six leaves per plant) of comparable age, georeferencing each plant to recognize them for further experiments into a natural patch of cotton plants of 2 ha into a the RL to 6.1 km from the nearest Town, the most distance between plants was 280 m. Genomic DNA was isolated from foliar tissue using the Miniprep CTAB method^2^. DNA concentrations were analyzed with a Quibit 3.0 Fluorometer (ThermoFisher Scientific, Massachusetts, USA). DNA sequences were amplified through a PCR endpoint, DNA polymerase GoTaq Flexi (Promega, USA), and the primers: Cry1Ab/Ac, Cry2Ab, and CP4EPSPS from Eurofins Scientific (Brussels, Belgium). The thermocycler was programmed for a denaturation period of 8 min at 95 °C, followed by 30 cycles of 20 s at 95 °C, 1 min at 60 °C, 1 min 72 °C, and then 8 min at 72 °C for the final cycle. Reactions were carried out in a volume of 25 µL containing 5 µL × buffer, 25 mM of magnesium chloride, 100 mM of dTTP, 10 µM primer, 70 nM of genomic DNA, and one unit of Taq polymerase (GoTaq Flexi by Promega). PCR products were separated in 2% denaturing agarose gel. We recorded the presence of three transgenes, *cry1ab/ac, cry2ab*, and *cp4epsps*, in individual and stacked form (Table 1). Based on the above, we combined in a single category all the plants that expressed the *cry* gene because they have the same insecticide effect. The initial sample size for our experiments of 61 plants was reduced to 21 plants, because the maximum amount of the W*cry* category was seven (Table 1). These plants were divided into three groups, with 7 individuals for each genotype (wild, introgressed with *cry*, and introgressed with *cp4-epsps*): (1) wild without transgenes, W (control); (2) wild with *cry*, W*cry*; and (3) wild with *cp4-epsps*, W*cp4-epsps.* For the purpose of this investigation we decided to exclude the plants expressing both transgenes, to avoid non additive effects. As described below, even with this limited amount of plants we were able to detect interesting results due to the genetic expression of these genes in a natural population, where plants coexist with their antagonists and mutualists. These results, of course, must be taken as a first line of evidence that requires further investigation with larger sample sizes.

**Table 1:** Percentage of plants found for each genotype (N = 61 plants).

Genotypes	Frequency of transgene (%)	Number of plants	Plants used in the experiments
W	39.34	24	7
W*cry* Insecticide effect	11.47	7*	7
W*cp4-epsps* Glyphosate tolerance	34.42	21	7
Combination of transgene *cry* and *cp4-epsps*	14.75	9	0

*The available plants of cry category were 7 whereby, this was our size sample per genotype.

### Experiment 1: effect of MeJA induction on extrafloral nectar production

Each experimental plant of the three genotypes was divided into two zones, in which we applied the control and induction treatments in the most distal branches^23^. Previously, we had evaluated whether induction through methyl jasmonate (MeJA) was active in cotton plants and found that indeed, cotton plants can respond to this elicitor by inducing extrafloral nectar in the branch where it is applied, hence the response was local and not systemic^21^. Considering this, control and induction treatments were applied in the same individual on different branches, using ten leaves that were sprayed with 2 mL of distilled water or MeJA solution (450 mM), respectively. To exclude insects from extrafloral nectaries, we coated plant branches with Tanglefoot in its original concentration (25% of natural gum resins) (Tangletrap, The Tanglefoot Corporation)^24^. Induction was carried out at 6:00 a.m. during three days of the summer of 2018 and concentration measurements of soluble solids were taken six hours later, at noon, based on previous observations on the peak of extrafloral nectar production. Nectar secretions from 10 leaves in each treatment(one nectary per leaf) per plant, in total 84 branches and 420 leaves were quantified; the amount of soluble solids was measured with 3 µL graduated micropipettes, and nectar concentration was obtained with a temperature-compensated portable refractometer (ATAGO hand refractometer, L Kübler, Karlsruhe, Germany). To remove the nectar, we applied 3 µL of distilled water to each nectary and repeated the procedure until we reached concentrations lower than 1%. Then, we added values from all collections in one leaf, to quantify leaf overall production of solid EFN compounds^23,25^.

### Experiment 2: effect of MeJA induction on abundance, richness, and composition of ants

After nectar measurements concluded, MeJA induction was carried out for two more days following the same experimental design as described above but allowing ant foraging. We conducted two censuses per day during a two-day period, at 10:00 a.m. and 4:00 p.m., when ant activity was at its highest (according to previous observations by our lab group). We registered the number of ants feeding and patrolling extrafloral nectaries and collected a sample of each ant species for taxonomic identification.

Ants were identified to species level using specialized publications or by comparison with specimens in the ant collection of the *Laboratorio de Invertebrados del Suelo* (INECOL, Xalapa, Mexico). Voucher specimens for all collected species were deposited in the latter collection (Table S2).

### Herbivore damage

We had to estimate the percentage of herbivore damage on the leaves, that is to say, the part of the leaves eaten by herbivores. Pictures of fresh leaves were captured (n = 10) for each branch of the genotypes described above. We used a Canon EOS Rebel T6i 18 MP camera without flash, positioned at 15 cm from the leaves placed above a scaled portable background. Pictures were taken in the field, preventing the leaves from being removed from the branches. Images were saved as JPG format and automatic quantification of leaf damage was performed with BioLeaf Analyze Foliar (version 1.0)^26^.

### Data analysis

Given that our data did not fit normal assumptions, our simples were analyzed using Generalized Linear Models with Quasipoisson distribution (GLM Ime4 package in R version 3.5.0), based on the dispersion of the data.The Poisson distribution and zero inflated models were also considered^27^. Consequently, we individually evaluated the effect of the induction treatment on EFN for each genotype using the Generalized Lineal Model with Quasipoisson distribution with the package GLM Ime4 in R version 3.5.0. Likewise, ant abundance and the percentage of herbivore damage were evaluated with Generalized Linear Models with Quasipoisson distribution (GLM Ime4 package in R version 3.5.0, where theinduction treatment (MeJA or control) and the genotype of the wild plants (W*cry*, W*cp4-esps*, and W) were considered as a fixed factor. We compared the effect of the induction treatment, genotype, date, and their interactions. Finally, ant richness and composition were expressed as the total of morphospecies per genotype. The scripts utilized for the analyses are available online at https://github.com/conservationgenetics/Ongoing-ecological-and-evolutionary-consequences-by-the-presence-of-transgenes-in-a-wild-cotton-popu.

## Results

### Effect of MeJA induction treatment on extrafloral nectar production in wild cotton with and without transgenes

**Figure 1:**
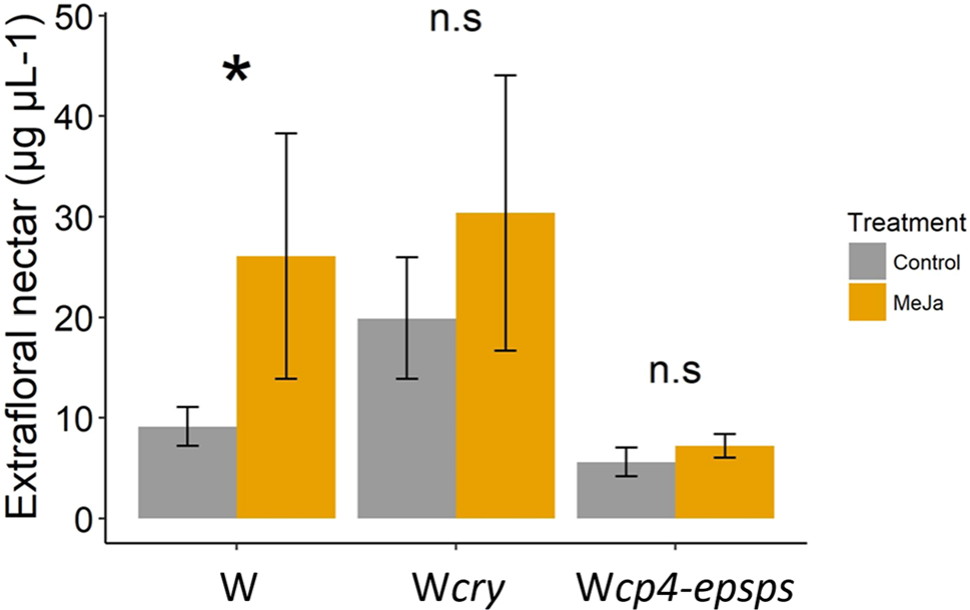
Effect of the induction treatment with MeJA on secretion of extrafloral nectar by wild *Gossypium hirsutum* plants without transgenes (W), wild with *cry* (W*cry*) and wild with *cp4-epsps* (W*cp4-epsps*) and within each plant. Grey bars represent the control treatment and yellow bars are the MeJA induction treatment. Values are means ± SE (N = 21, n = 84). The asterisk (*) indicates significant differences according to a post-hoc test.

Exogenous application of MeJA significantly increased EFN secretion in wild plants without transgenes (W) (*P*(χ^2^) = 0.048, *df* = 1). In contrast, transgenic genotypes (W*cry* and W*cp4-epsps)* did not respond to this stimulus (W*cry: P*(χ^2^) = 0.375, *df* = 1; W*cp4-epsps*: *P*(χ^2^) = 0.388, *df* = 1), because of different reasons regarding the genotypes with introgressed genes. Whereas W*cry* control plants had a high EFN production equivalent to the induced state of W plants, EFN production in W*cp4-epsps* plants was inhibited, yielding less than 8 µg/mL in both treatments (Fig. 1). When comparing the control response between genotypes, the extrafloral production in the W*cry* genotype was significantly different to the one found in W and W*cp4-epsps* (*P*(χ^2^) < 0.001, *df* = 2) (Fig. 1). And, when comparing the induction response between induced and control treatment of the W and W*cry* genotypes, respectively, we observed no differences (*P*(χ^2^) = 0.637, *df* = 1).

### Abundance, richness and composition of ants associated with cotton’s EFN

We collected 109 ants associated with the EFN of *G. hirsutum,* which belonged to eight species. The highest abundance and richness was registered in the W genotype (42 ants and 7 species), meanwhile in W*cry* and W*cp4-epsps* we registered 33 and 34 ants, belonging to four and six species, respectively (Table 2). Four species of ants displayed defensive behaviour: *Camponotus planatus, Camponotus rectangularis aulicus, Dorymyrmex bicolor*, and *Pseudomyrmex gracilis*. Likewise, we registered two ant species without defensive behaviour associated with the wild cotton' EFN, and one of these is invasive (*Paratrechina longicornis*).

**Table 2:** Ant composition and abundance associated with wild cotton plants.

Ant species	W		W*cry*		W*cp4-epsps*	
	Control	MeJA	Control	MeJA	Control	MeJA
*Paratrechina longicornis*	6	8	–	2	1	1
*Camponotus planatus*	8	14	3	20	1	5
*Camponotus rectangularis aulicus*	1	–	1	5	4	3
*Pseudomyrmex gracilis*	1	–	2	–	3	–
*Brachymyrmex* sp*.*	2	–	–	–	–	–
*Dorymyrmex bicolor*	–	1	–	–	1	6
*Temnothorax subditivus*	1	–	–	–	–	–
*Monomorium ebeninum*	–	–	–	–	7	2

The ant' community composition associated with cotton' EFN was different between genotypes. W plants had two exclusive species, W*cp4-epsps* had an exclusive ant species, while W*cry* didn't have exclusives ants, and four of the ants collected were presented in all genotypes (Table 2).

### Effect of MeJA induction treatment on the abundance of ants in wild cotton with and without transgenes

**Figure 2:**
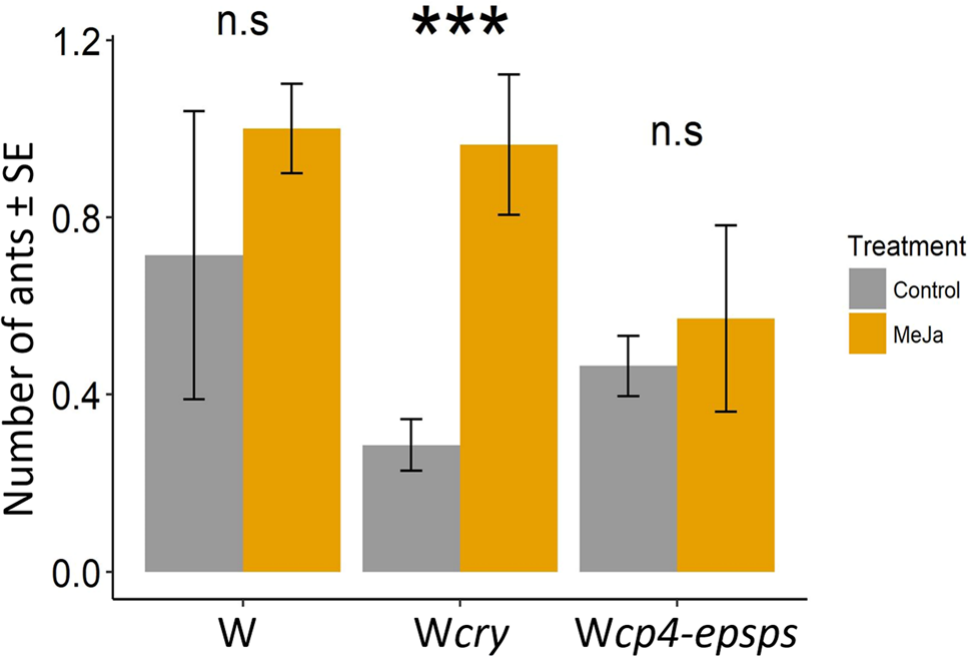
Effect of induction treatment on ant abundance in three genotypes of wild *Gossypium hirsutum* L.: wild cotton without transgenes (W), wild with *cry* (W*cry*), and wild with *cp4-epsps* (W*cp4-epsps*). Grey bars are for the untreated control and yellow bars for MeJA (induction treatment). Bars show means ± SE (N = 21 n = 42). n.s. indicating non-significant differences, and *** indicates significant differences (*P* < 0.001) according to the Tukey post-hoc test between constitutive and induced conditions.

Exogenous application of MeJA significantly affected the abundance of ants in the W*cry* genotype (*P*(χ^2^) < 0.001, *df* = 1), with *C. planatus* being the most abundant ant species. In contrast, in the W and W*cp4-epsps* genotypes the induction treatment did not have any effect on ant abundance (W: *P*(χ^2^) = 0.436, *df* = 1; W*cp4-epsps*: (*P*(χ^2^) = 0.210, *df* = 1) (Fig. 2).

### Herbivore damage in wild cotton with and without transgenes

**Figure 3:**
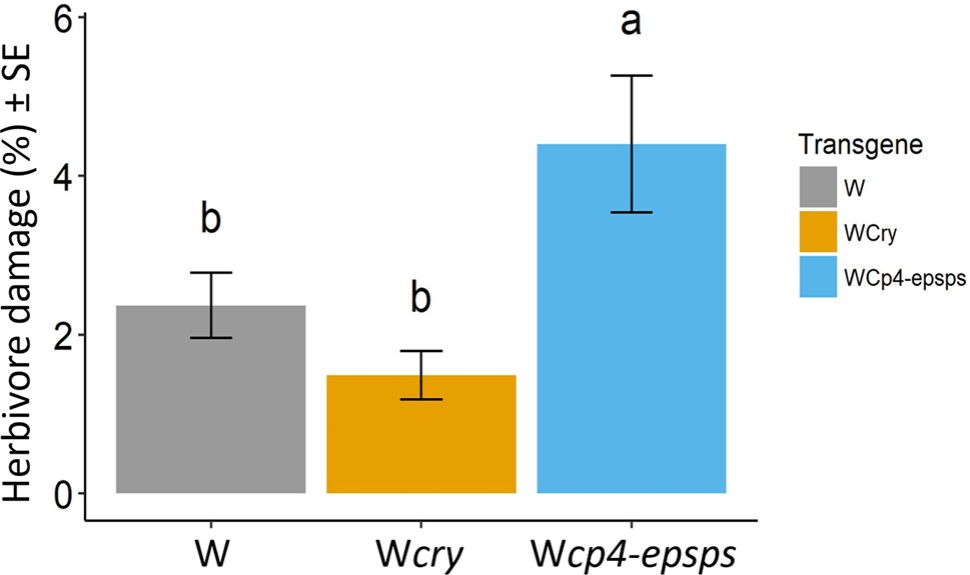
Herbivore damage in three genotypes of wild *Gossypium hirsutum* L: wild cotton (W; grey), wild with *cry* (W*cry*; yellow), and wild with *cp4-epsps* (W*cp4-epsps*; blue). Bars show means ± SE (N = 21, n = 84). Different letters indicate significant differences according to Tukey *Post-hoc* test.

W*cp4-epsps* was the genotype with the highest herbivore damage value that was significantly different (*P*(χ^2^) < 0.001, *df* = 2) when compared to W and W*cry* genotypes. The lowest percentage of herbivore damage value was registered for W*cry* plants (Fig. 3).

## Discussion and conclusions

In this study, we showed that the expression of *cry* and *cp4-epsps* genes in wild cotton altered the secretion of EFN, the associations with different ant species, and the levels of herbivore damage on target plants. W*cry* constantly maintained a high production of EFN, regardless of the MeJA treatment, but nectar production was minimal in W*cp4-epsps*. These changes in nectar inducibility seem to modify the composition of ant communities, foster the dominance of the generalist and defensive species *C. planatus* in *Bt* plants and the presence of ants without defensive role, *M. ebeninum*, in the herbicide tolerant genotype, while W plants had both defending species (*C. planatus, C. rectangularis aulicus* and *P. gracilis*) and invasive ant species (*P. longicornis*) in the same proportion. Furthermore, herbivore damage and its associated ant community were different according to the introgressed transgene.

### Wild and introgressed cotton do not display phenotypic equivalence in natural conditions

In general, it has been assumed that introgressed and wild genotypes should display similar phenotypes in the absence of the selection agents targeted by transgenes. However, when we compared the control group and the three genotypes, we registered different nectar secretion patterns among them (Fig. 1). Similar results have been registered in populations of *bt* rice and glyphosate-tolerant sunflowers living in natural conditions where introgressed plants are different from their wild relatives^5^.

### Transgene expression modified indirect induced defences in wild cotton

Most plants are able to induce responses after herbivore damage and/or phytohormone exogenous application (i.e. jasmonic acid, JA; methyl jasmonate, MeJA; and salicylic acid, SA)^11,28,29^. However, unlike wild plants without transgenes, individuals with transgenes were not sensitive to the induction treatment with MeJA for increasing their EFN production (Fig. 1). These results contrast with previous reports on cultivated varieties, such as *Bt* and glyphosate-resistant (c*p4-epsps*), in which direct defences such as gossypol terpenoids (160%), hemigossypolone (160%), helicoids 1|4 (213%) and indirect defenses, such as volatile compounds (VOCs) (171.2%) and extrafloral nectar (EFN) (133%), were reported to increase in plants sprinkled with JA and MeJA^21,28–30^.

The inability of plants with transgenes to have the production of extrafloral nectar induced in them was related to different processes dependent on the identity of the transgenes in question. Whereas W*cry* control plants had a high EFN production equivalent to the induced state of W plants, EFN production in W*cp4-epsps* plants was inhibited. Contrasting these findings with results obtained under controlled conditions (i.e. greenhouse and crop conditions)^3,21^, we suggest that EFN production is linked to genotypes with transgenes and abiotic stress in the coastal dunes, because transgenes are connected to main metabolic pathways that respond to stressful conditions^21^*.*

### Wild cotton with *cp4-epsps*

**Figure 4:**
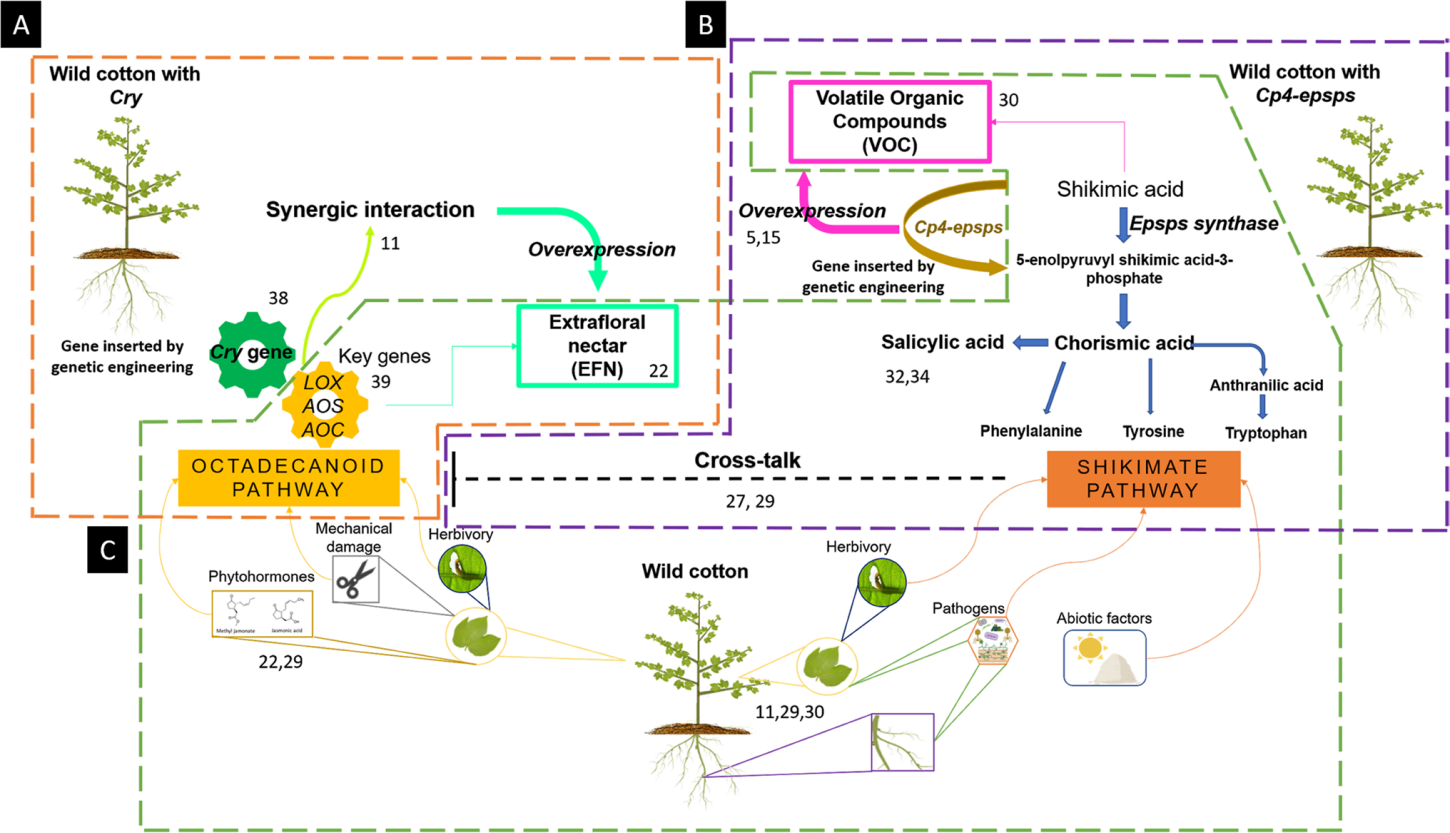
A diagram illustrating how the expression of *cry* (**A**) and *cp4-epsps* (**B**) in absence of their selection agent (pests and glyphosate) can affect the extrafloral nectar production. The extrafloral nectar (EFN) production is an induced defence that can be triggered by foliar herbivory, mechanical damage, and exogenous application of phytohormones (i.e. jasmonic acid, methyl jasmonate, and salicylic acid). These factors activate the octadecanoid pathway, and therefore, the production of extrafloral nectar, (**A**) aqua rectangle. The (**C**) section is an example of this reaction in a wild cotton plant (without transgenes). After damage, the key genes (yellow mesh) of the octadecanoid pathway are activated and produce extrafloral nectar. Another scenario is when the wild cotton expresses *cry* genes (**A** section), in this case, the key genes of the octadecanoid pathway interact synergistically with the *cry* transgene (green mesh). This triggers an over-expression of the production of EFN (aqua thick arrow), switching from inducible to constitutive responses. When the plants express *cp4-epsps* (**B** section), the production of extrafloral nectar is reduced or inhibited. A possible answer is an over-expression of the *epsps* gene (gold curve arrow), that increased production of salicylic acid which creates a crosstalk between shikimate and octadecanoid pathways (black cross-talk arrow). When the shikimate pathway is activated, the principal inducible defence is the production of volatile organic compounds (VOCs) (pink rectangle).

In the absence of herbicides acting as a selection agent, wild plants with *cp4-epsps* exhibited large differences compared to wild plants without them. Their low nectar production (> 8 µg/mL) (Fig. 1) could be linked to the crosstalk between the jasmonate and the salicylate (SA) pathways (Fig. 4, orange and purple section). In *G. hirsutum* and other species, SA signalling has been proven to negatively affect JA signalling (e.g. *Zea mays*, *Solanum lycopersicum*, *Nicotiana tabacum* and *Arabidopsis thaliana*)^31–33^: therefore, we suggest an interference between the SA and JA pathways given previous reports that an over-expression of the c*p4-epsps* gene modifies the second part of the shikimate pathway (post-chorismate), which leads to the synthesis of essential amino acids as phenylalanine, tryptophan, or tyrosine, the latter being a precursor of benzoic acid BE, and SA^34,35^ (Fig. 4, purple section). This evidence highlights that hidden crosstalk effects among different metabolic pathways can scale up and modify plant phenotypes (e.g. extrafloral nectar production).

### Wild cotton with *cry*

Wild cotton plants with *cry* genes continuously produced EFN as a constitutive defence (Fig. 1), in equivalent quantities as the induced state of W plants. EFN production is regulated by the octadecanoid signalling pathway, which can be activated by herbivore damage, mechanical damage, and phytohormones, such as JA and MeJA^21,28^ (Fig. 4, green section). However, for cotton, a specific elicitor is not necessary^36^. Four key genes for the synthesis of JA and MeJA have been described: *AOS*, *AOC*, *HPL*, and *COI1*^37^. In *Bt* maize, studies comparing GM corn and its isogenic lines report an increase of 24% in phenols and 63% of DIMBOA (2,4-dihidroxi-7-metoxi-1,4-benzoxazin-3-ona; natural defences against lepidopteran herbivores)^11^. This is consistent with observations of a synergy between maize direct defences and *Bt* genes, after exogenous applications of JA (Fig. 4, orange section). Considering the latter, we suggest that W*cry* cotton may present a similar response, as the genes activating the JA pathway are *GhAOS* and *GhCOI1* (homologs to maize JA biosynthesis genes: *ZmAOS* and *ZmCOI1*), in addition to *Ghppo1*, which confers natural resistance to lepidopteran pest, such as *H. armigera*^38^. The interaction of *cry* with other genes could modify the production of EFN in W*cry* plants.

### Effect of the transgenes’ expression on ants associated to wild cotton

We identified eight species of ants harvesting EFN (Table 2), but with distinctive communities as a function of the plant genotype. This result suggests that the change in quantity, and possibly the composition and quality of EFN, can influence the ant community associated with *G. hirsutum*^39–41^.

Changes in plant reward production could potentially compromise the attraction of natural enemies of herbivores^42^. In our study, the availability of EFN was modified. Although species richness was the same as in W plants (Table 2), the most abundant ant species associated with W*cp4-epsps* plants, *M. ebeninum,* is considered a generalist species. Moreover, due to the lack of aggressive behaviour, this species does not represent an effective biotic defence^43^. The high abundance of this non-defensive species could be associated with the greater herbivore damage observed in W*cp4-epsps* plants (Fig. 2). In contrast, W or W*cry* plants showed a greater abundance of more aggressive ant species such as *C. planatus*, *C. rectangulatus*, and *P. brunneus* and significantly less herbivore damage.

In W*cry* cotton, the community of patrolling ants was mainly dominated by *C. planatus*, in both treatments (control and induction). Interestingly, although the amount of nectar did not vary between treatments, the abundance of ants was significantly different. The dominance of a single ant species could have benefited the plants with increased indirect defence, reducing herbivore damage and promoting a greater seed production per plant, as described in *Turnera ulmifolia*^44^*, Schomburgkia tibicinis*^45^, and *Opuntia stricta*^42^. However, considering the aggressive and dominant behaviour of *C. planatus*, there may be ecological costs through antagonistic relationships with pollinators. Ants can interrupt pollination and affect plant fitness^25,46,47^. The outcome of these mutualistic and antagonistic interactions requires further study.

### Effects of transgenes on herbivore damage

Considering that the type of mutualism that cotton sustains with ants is defensive, we suggest that the change we observed in the composition of ants is likely to have influenced herbivore damage in the different genotypes, which in turn has the potential to reduce fitness as shown by other studies of cotton^48–50^. However, a study carried out on wild upland cotton reported that plants tolerate intermediate levels of leaf damage inflicted by leaf-chewing insects (< 50%)^51^. Thus, the increased herbivore damage observed in W*cp4-epsps* plants (< 10%; 4.402 ± 0.863) does not necessarily jeopardize plant growth or reproductive success. For example, in the absence of glyphosate, transgenic hybrids of rice and soybean show changes in their phenology (i.e. early flowering and shorter germination times) and an increase in their fitness (i.e. larger fruit and seed sets) compared to their wild relatives^4,5^. It has been suggested that the over-expression of the *epsps* gene is responsible for these compensation effects on plant fitness, in view of the biotic and abiotic stressors that activate the shikimate pathway^5,15^. Further investigation to find out if this is the case for W*cp4-epsps* cotton is currently being developed under natural conditions.

In contrast, W*cry* plants exhibited the lowest herbivore damage, although not significantly different from wild plants without transgenes (Fig. 3). The expression of *cry* genes, conferring a new defensive trait against lepidopterans, may represent an advantage for plants under selection when target herbivores are present. For example, within the natural distribution of wild sunflowers, genotypes with *cry* genes showed less herbivore damage than their wild relatives (WR), increasing their seed production by 55%^4^. Nonetheless, in *G. hirsutum* we did not find differences in herbivore damage when compared to the W and W*cry* genotypes, probably because lepidopteran species targeted by *Bt* cotton were not present in our study site, hence, *cry* expression might not have a role in increasing fitness. However, indirect effects could be present through its interaction with native herbivores. For example, experiments under laboratory and experimental conditions conducted on maize with *cry* genes, have shown non-lethal effects in the physical condition of non-target caterpillars (e.g. smaller size, lower weight, lower survival, and more larval instars)^52^. Likewise, if the nutritious quality of herbivore tissues changes after consuming *Bt* toxins, these effects could cascade to higher trophic levels^53,54^, increasing mortality and decreasing longevity or development of predators. This has been shown for chewing predators (e.g. *Chrysoperla carnea* and lady beetles, Coccinellidae) because they ingest the gut of the prey, where most of the toxins are concentrated^54^. Parasitoids have also been found to be affected after the consumption of *Bt* toxins contained in their preys^55^. When the wasp *Microplitis mediator* (parasitoid of *H. armigera*) was fed with larvae containing *cry* toxins, they extended its egg and larval development time by 1–2 days, significantly decreasing its pupal weight by 35%, and its overall longevity when the toxin concentrations were high (4–8 μg g^−1^). Hence, a question that warrants further investigation is how W*cry* cotton genotypes in the wild affect herbivore and predator communities and their interactions.

By means of an integrative methodology, we evaluated the effect of the *cry* and *cp4-epsps* gene expression in wild cotton plants. As a result, we obtained the following noteworthy results: (1) differential response in the induced defence mechanism (extrafloral nectar production) concordat to plant genotype; and (2) modification of biotic interactions between introgressed cotton and relevant organisms, under natural conditions.

Although several hypotheses have been raised regarding the consequences of GMO release into the environment (e.g. gene flow, hybridization, and introgression), these have only been tested under controlled conditions. We found that some theory-based concerns can be confirmed when performing functional experimental designs in the wild. First, it is possible to investigate ecological and evolutionary impacts of new genes under natural conditions by studying community processes, such as changes in tri-trophic interactions. Second, we detected physiological and possible metabolic alterations generated by the expression of transgenes in wild cotton plants without the pressure of selection agents (pests and herbicides) targeted by those genes. Third, until 2008, 4 wild metapopulations of upland cotton showed evidence of introgression with GM cotton^2^, and recently, introgression in the Baja California Sur, BCSM, Central Pacific, CPM, and Yucatan Peninsula YPM metapopulations has been reported^3^. In this study, we reaffirm the presence of transgenes in the YPM, but it's important to consider that the establishment of these has been fast. Whereas during the first monitoring in 2008, YPM metapopulation didn't register the presence of transgenes, in 2018, 60.64% of 61 plants had them (Table 1).

In natural ecosystems, changes at different scales (i.e. genetic, individual, and community), following the introduction of novel genes lead to endless research possibilities. Through them we can integrate broad information regarding biological control, agro-biotechnologies, and conservation biology, with promising further applications. Although we do not know the routes of transgene dispersion, we provide evidence of some of the mechanisms that could favour the establishment and persistence of these new genes, mainly due to their interaction with key defence metabolic pathways. However, transgene frequency in wild populations and the associated ecological consequences must continue to be monitored and evaluated so as to contribute information that allows us to make decisions for the conservation of the primary genetic pool and the ecological and evolutionary processes that have shaped its diversity.

Alterations in the defence mechanisms of wild relatives in one of the most important crops for humanity represents critical evidence on the threats of introgressed genes to biological and cultural heritage for the following generations. Such negative consequences would be enough to envision GMO liberation into the environment, short and long distance from its origin centre, from a different perspective. At this point, we are in a watershed moment to: (1) develop the necessary research to mitigate the ecological, evolutionary caused by the introgression of GMOs into the wild-to-domesticated complex of the species used, as well as reformulating risk assessments for protection goals; (2) fill gaps in our knowledge of the complex dispersion routes of transgenes from crop to wild relatives and native varieties; and finally, (3) prioritize the *in-situ* conservation of the primary gene pool without transgenes. All these efforts would contribute to the integration of the available disperse information so we may understand the consequences of transgenic plants coexisting with their relatives. As we demonstrated in the current study, the presence of these genes can cause intrinsic changes in wild populations of cotton (allelic frequency), and changes in their ecological interactions. If we want to conserve *in-situ* the primary gene pool of wild relatives, we must work to identify the ecological and evolutionary processes affected by the existence and permanence of these transgenes within their populations. Upon the detection of these genes, mitigation strategies to reduce the magnitude of the damage can be promptly designed.

## Supplementary Information

Supplementary Table S1.

Supplementary Table S2.
