# Supplementary material for: Ongoing ecological and evolutionary consequences by the presence of transgenes in a wild cotton population
Source: Sci Rep. 2021 Jan 21;11:1959. doi: 10.1038/s41598-021-81567-z (PMC7820435; doi:10.1038/s41598-021-81567-z)
Supplement: Supplementary file 1 — Supplementary Table S1. [file 41598_2021_81567_MOESM1_ESM.docx]

Supplementary information for: Ongoing ecological and evolutionary consequences by the presence of transgenes in a wild cotton population

Valeria Vázquez-Barrios^1,2^, Karina Boege^3^, Tania Gabriela Sosa-Fuentes^2^, Patricia Rojas^4^ & Ana Wegier^2^*

^1^ Posgrado en Ciencias Biológicas, Instituto de Biología, Universidad Nacional Autónoma de México, Mexico City, Mexico.

^2^ Laboratorio de Genética de la Conservación, Jardín Botánico, Instituto de Biología, Universidad Nacional Autónoma de México, Mexico City, Mexico.

^3^ Departamento de Ecología Evolutiva, Instituto de Ecología, Universidad Nacional Autónoma de México, Mexico City, Mexico.

^4^ Red de Biodiversidad y Sistemática, Instituto de Ecología A.C., Xalapa, Veracruz, Mexico.

*correspondence author: awegier@ib.unam.mx

Supplementary table 1: References material

The cotton plants used this study were identified to species level by comparing with references material from the Herbario Nacional de México (MEXU) of the same location.

Table S2. *Gossypium hirsutum* plants comparative material of Herbario Nacional de México (MEXU)

| Collection | ID | Catalogue number | Scientific name reference | Date | Colection number | Collector | Location |
| --- | --- | --- | --- | --- | --- | --- | --- |
| Herbario Nacional de México (MEXU) | IBUNAM:MEXU:781795 | 781795 | THEPLANTLIST:kew-2831092 | 06/09/1979 | 2903 | H. Quero, H. Flores y R. Grether | Las coloradas, Yucatan, Mexico |
| Herbario Nacional de México (MEXU) | IBUNAM:MEXU:984966 | 984966 | THEPLANTLIST:kew-2831092 | 31/08/1991 | 279 | M. Méndez, R. Durán y J. Granados | Las coloradas, Yucatan, Mexico |
| Herbario Nacional de México (MEXU) | IBUNAM:MEXU:512439 | 512439 | THEPLANTLIST:kew-2831092 | 20/12/1985 | 10094 | E. Cabrera con H. de Cabrera | Ría Lagartos, Yucatan, Mexico |
