# Supplementary material for: Ongoing ecological and evolutionary consequences by the presence of transgenes in a wild cotton population
Source: Sci Rep. 2021 Jan 21;11:1959. doi: 10.1038/s41598-021-81567-z (PMC7820435; doi:10.1038/s41598-021-81567-z)
Supplement: Supplementary file 2 — Supplementary Table S2. [file 41598_2021_81567_MOESM2_ESM.docx]

Supplementary information for: Ongoing ecological and evolutionary consequences by the presence of transgenes in a wild cotton population

Valeria Vázquez-Barrios^1,2^, Karina Boege^3^, Tania Gabriela Sosa-Fuentes^2^, Patricia Rojas^4^ & Ana Wegier^2^*

^1^ Posgrado en Ciencias Biológicas, Instituto de Biología, Universidad Nacional Autónoma de México, Mexico City, Mexico.

^2^ Laboratorio de Genética de la Conservación, Jardín Botánico, Instituto de Biología, Universidad Nacional Autónoma de México, Mexico City, Mexico.

^3^ Departamento de Ecología Evolutiva, Instituto de Ecología, Universidad Nacional Autónoma de México, Mexico City, Mexico.

^4^ Red de Biodiversidad y Sistemática, Instituto de Ecología A.C., Xalapa, Veracruz, Mexico.

*correspondence author: awegier@ib.unam.mx

Supplementary table 2: Ants species

The ants collected for this study were identified to species level using specialized publications or by comparing with references material from the ant collections of Laboratorio de Invertebados del Suelo of Instituto de Ecología (INECOL, Xalapa). Voucher specimens of all species were deposited in latter Collection.

| Table S1. Ants species identified and collection number of ant collection of Laboratorio de Invertebrados del Suelo, INECOL, Xalapa | |
| --- | --- |
| Ants species | Voucher specimens |
| *Paratrechina longicornis* | BSIE8273, BSIE8274, BSIE8275 |
| *Camponotus planatus* | BSIE8276, BSIE8277, BSIE8278, BSIE8279, BSIE8280, BSIE8281, BSIE8282, BSIE8283 |
| *Camponotus rectangularis aulicus* | BSIE8284, BSIE8285, BSIE8286, BSIE8287, BSIE8288 |
| *Pseudomyrmex gracilis* | BSIE8289, BSIE8290, BSIE8291, BSIE8292, BSIE8293 |
| *Brachymyrmex sp.* | BSIE8294 |
| *Dorymyrmex bicolor* | BSIE8295, BSIE8296 |
| *Temnothorax subditivus* | BSIE8297 |
| *Monomorium ebeninum* | BSIE8298, BSIE8299, BSIE8300 |
